# An Eight-Channel C-Band Demux Based on Multicore Photonic Crystal Fiber

**DOI:** 10.3390/nano8100845

**Published:** 2018-10-17

**Authors:** Dror Malka, Gilad Katz

**Affiliations:** Faculty of Engineering, Holon Institute of Technology (HIT), Holon 5810201, Israel; giladka@hit.ac.il

**Keywords:** photonic crystal fiber, demultiplexer, dense wavelength division multiplexing

## Abstract

A novel eight-channel demux device based on multicore photonic crystal fiber (PCF) structures that operate in the C-band range (1530–1565 nm) has been demonstrated. The PCF demux design is based on replacing some air-hole areas with lithium niobate and silicon nitride materials over the PCF axis alongside with the appropriate optimizations of the PCF structure. The beam propagation method (BPM) combined with Matlab codes was used to model the demux device and optimize the geometrical parameters of the PCF structure. The simulation results showed that the eight-channel demux can be demultiplexing after light propagation of 5 cm with a large bandwidth (4.03–4.69 nm) and cross-talk (−16.88 to −15.93 dB). Thus, the proposed device has great potential to be integrated into dense wavelength division multiplexing (DWDM) technology for increasing performances in networking systems.

## 1. Introduction

Dense wavelength division multiplexing (DWDM) is a system [1,2] that is used to integrate information from different sources over one fiber, while each source carried on its own divided light wavelength at the same time. DWDM has the ability to divide sources up into 80 ports, and allow more information to be multiplexed into a light-stream that is transferred on one fiber. Demux is an essential device in the DWDM system, and its main functionality is to divide signals from one input port into multiple ports. The demux device has several advantages such as a low bit error rate [3], a high data rate, a large bandwidth, low cross-talk [4], and less propagation delay. Therefore, researchers have shown the potential of designing demux-based waveguide techniques such as silicon photonics [5], Y-branch [6], multimode interference (MMI) [7,8,9], Mach–Zehnder interferometers [10,11], MMI in slot waveguide structures [12,13,14], etc. 

Photonic crystal fiber (PCF) is a powerful waveguide that is based on a microstructured arrangement of materials of different refractive indexes [15]. The background material is usually pure silica, and the low-index regions are air-holes that are located along the fiber length. Several works have demonstrated the great potential of using PCF structures in comparison to conventional fibers [16,17,18]. The main benefit of designing a demux device based on a PCF structure is its ability to integrate different materials that have a high difference in their refractive index values. This is because the light guiding mechanism in PCF is based on the bandgap and modified total internal reflection (MTIR). Another advance is the ability to achieve a lower coupling length, especially in the case of closer coupled ports (cores) [19]. 

Several techniques have demonstrated how it is possible to couple light between closer coupled ports (cores) in a PCF structure through methods such as changing the PCF index profile by replacing some air-hole regions with pure silica along the fiber length [19,20] and using different air-hole sizes in the PCF structure [21,22,23,24]. 

The C-band range is set from 1530 nm to 1565 nm in the wavelength, and it is the most efficient and useful range in the optical communication field [14]. The advance of the C-band is the ability to transmit data with a high bitrate over a long distance since the C-band supports DWDM and optical amplifier fiber technologies [25]. 

In this work, we demonstrated a 1 × 8 wavelength demux in a PCF structure that split eight wavelengths in the C-band range. The operating wavelengths are between 1530–1565 nm with a spacing of 5 nm between two wavelengths. The light coupling between the closer coupled cores was obtained by replacing some air-hole areas with lithium niobate (LiNbO_3_) and silicon nitride (Si_3_N_4_) materials along the fiber length. 

Numerical investigations were carried out on the locations of the LiNbO_3_ and Si_3_N_4_s layers and the key geometrical parameters of the multicore PCF structure to obtain high efficiency demultiplexing between the operating wavelengths. The demux PCF structure was analyzed and simulated using a beam propagation method (BPM) and Matlab codes. This device can be useful to increase the data bitrate in the C-band using a DWDM system. 

To the best of our knowledge, this paper is the first to study the controlling of light propagation direction by integrating LiNbO_3_ and Si_3_N_4_ rods over a multicore PCF structure. This new study was utilized to design a new demultiplexer based on a multicore PCF structure. Thus, the novelty is that the demultiplexer operated within the multicore PCF structure without using additional optical components. This can lead to a new DWDM technique that can be utilized to reduce the costs and size of the optical communication system.

## 2. Materials and Methods

**Figure 1:**
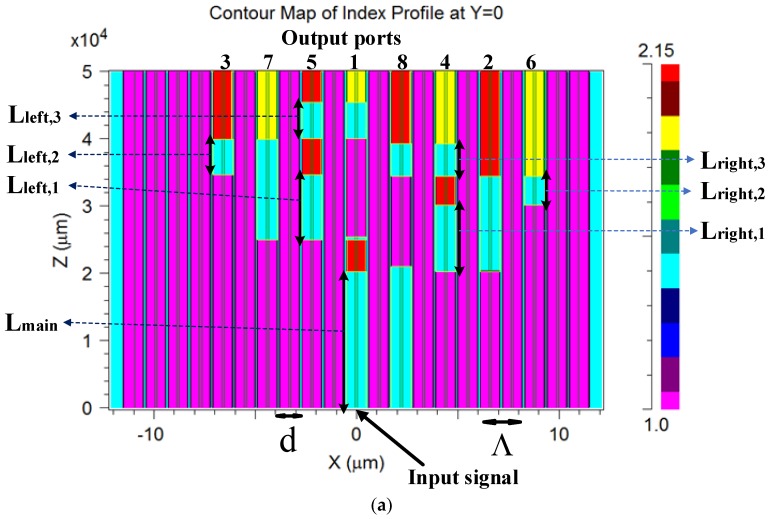

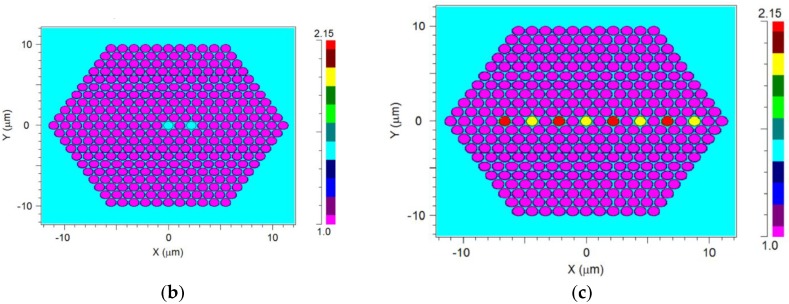
Refractive index profile of the 1 × 8 wavelength demux: (**a**) XZ plane at y = 0 cm. (**b**) XY plane at z = 0 cm. (**c**) XY plane at z = 5 cm.

Figure 1a–c show the full refractive index profile structure of the demux PCF design on the XZ plane (y = 0 cm), XY plane (z = 0 cm), and XY plane (z = 5 cm), respectively. In these figures, the background material is pure silica, and it is marked in a light blue color; the air-hole areas are marked in a purple color, the LiNbO_3_ areas are marked in a red color, and the Si_3_N_4_ areas are marked in a yellow color. The geometrical parameters of the PCF structure are d, Λ (pitch), and z. In Figure 1a,d represents the hole diameter of the air-holes, pitch represents the distance between two air-holes, and z represents the light propagation axis.

The principle work of the PCF demux device is based on light confinement inside the channel (core) and controlling the light coupling length size between two closer ports (cores). These effects can be obtained by replacing some air-hole regions with a high-index material along the fiber length. Furthermore, the coupling length value can be shifted by changing the operating wavelength. Thus, the suitable coupling length value between two closer ports can be found by optimized the PCF geometrical parameters (z, Λ, d) and the materials’ layer locations over the light propagation axis (z).

In our design, the light-guiding mechanism is based on the bandgap and the MTIR, which means that light can be coupled between two closer ports that have the same refractive index value (light blue color areas in Figure 1a), and a strong light confinement can be obtained by a port that has a high index value (red and yellow areas in Figure 1a).

It can be noticed from Figure 1a that the areas in which light can be coupled between closer silica (light blue color) ports (cores) are located as follows: port 1 and port 8 over the Z-axis range of 0–2.03 cm (L_main_), port 8 and port 4 over the Z-axis ranges of 2.03–2.1 cm and 3.445–3.93 cm (L_right_,_3_), port 1 and port 5 over the Z-axis range of 2.5–2.545 cm and 4–4.545 cm (L_left,3_), port 4 and port 2 over Z-axis range of 2.03–3.01 cm (L_right_,_1_), port 5 and port 7 over the Z-axis range of 2.5–3.46 cm (L_left,1_), port 2 and port 6 over the Z-axis range of 3.01–3.44 cm (L_right_,_2_), port 3 and port 7 over the Z-axis range of 3.47–3.995 cm (L_left,2_). In addition, it can be noticed that each port at the output has a light confinement area, as shown in Figure 1a. Figure 1b,c show the input port at z = 0 (port 1) and eight output ports at z = 5 cm (red and yellow colors).

Table 1 shows the refractive index material values for the C-band range [26,27,28], and it is important to mention that the Si_3_N_4_ and LiNbO_3_ materials have a very low absorption in this range. Another advance to use these materials is that light cannot be coupled between closer Si_3_N_4_ and LiNbO_3_ layers, because they have a small difference in the index value, as shown in Table 1.

The Si_3_N_4_ and the LiNbO_3_ layers were used to enable a strong light confinement inside the port that ensures that light cannot be coupled to other closer ports. In the proposed design, there are 11 layers that function as follows: eight output lines (four LiNbO_3_ layers for ports 2, 3, 5, and 8, and four Si_3_N_4_ layers for ports 1, 4, 6, and 7) and three selected lines in a similar way to the classical definitions of the digital 1 × 8 demultiplexer. 

The three LiNbO_3_ layers were used to function as the selected waveguide switch that controls the light propagation direction along the fiber length. The first selected switch is located at the Z-axis range between 2.03–2.5 cm, and can demultiplex the light propagation between ports 1, 5, 7, and 3, and ports 8, 4, 2, and 6. The second selected switch is located at the Z-axis range between 3.01–3.445 cm, and can demultiplex the light propagation between ports 8 and 4, and ports 2 and 6. The third selected switch is located at the Z-axis range between 3.46–4 cm, and can demultiplex the light propagation between ports 3 and 7 and ports 5 and 1.

It is important to emphasize that the coupling length of the resonated light between two closer ports is dependent on the key geometrical parameters (d, Λ) and the operating wavelength. Thus, the coupling length can be found using the equation below [29,30]:

(1) $$L^{\lambda }{}_{Coupling}=\frac{\pi }{k_{0}\left(n_{symmetric}\left(d,\Lambda ,\lambda \right)-n_{anti-symmertric}\left(d,\Lambda ,\lambda \right)\right)}\;$$

where k_0_ is the free-space wave vector, λ is the operating wavelength, and n_anti-symmetric_ and n_symmetric_ are the anti-symmetric and symmetric effective refractive index, respectively.

It is worth mentioning that the coupling length is periodical due to the oscillation between two closer ports. 

The conditions for divide eight different wavelengths in the proposed design are given by:

(2) $$\begin{array}{l} L_{main}\approx p_{1}L_{Coupling}^{\lambda_{1},\lambda_{3},\lambda_{5},\lambda_{7}}=(p_{1}+q_{1})L_{Coupling}^{\lambda_{2},\lambda_{4},\lambda_{6},\lambda_{8}}\\ L_{right,1}\approx p_{2}L_{Coupling}^{\lambda_{8,},\lambda_{4}}=(p_{2}+q_{2})L_{Coupling}^{\lambda_{2,}\lambda_{6}}\\ L_{left}{}_{,1}\approx p_{3}L_{Coupling}^{\lambda_{3,}\lambda_{7}}=(p_{3}+q_{3})L_{Coupling}^{\lambda_{5,}\lambda_{1}}\\ L_{right,2}\approx p_{4}L_{Coupling}^{\lambda_{2}}=(p_{4}+q_{4})L_{Coupling}^{\lambda_{6}}\\ L_{left}{}_{,2}\approx p_{5}L_{Coupling}^{\lambda_{3}}=(p_{5}+q_{5})L_{Coupling}^{\lambda_{7}}\\ L_{right,3}\approx p_{6}L_{Coupling}^{\lambda_{8}}=(p_{6}+q_{6})L_{Coupling}^{\lambda_{4}}\\ L_{left}{}_{,3}\approx p_{7}L_{Coupling}^{\lambda_{5}}=(p_{7}+q_{7})L_{Coupling}^{\lambda_{1}} \end{array}$$

where L_main_ is the fiber length located between core 1 and core 8 (silica areas), L_righ,1_ is the fiber length located between core 2 and core 4 (silica areas), L_left,1_ is the fiber length located between core 7 and core 5 (silica areas), L_righ,2_ is the fiber length located between core 2 and core 6 (silica areas), L_left,2_ is the fiber length located between core 3 and core 7 (silica areas), L_righ,3_ is the fiber length located between core 8 and core 4 (silica areas), L_left,3_ is the fiber length located between core 5 and core 1 (silica areas), p_1/2/3/4/5/6/7_ is a natural number, and q_1/2/3/4/5/6/7_ is an odd number. 

For analyzing the performances of our proposed 1 × 8 wavelength demultiplexer multicore PCF, cross-talk (Equation (3)) and loss (Equation (4)) were calculated to observe the ratio between a desirable and undesirable wavelength in a given port and power losses, respectively.

(3) $$C.T_{n}=\frac{1}{3}\sum_{m=1}^{4}10\log\left(\frac{P_{m}}{P_{n}}\right)\;$$

where P_n_ is the power transmission for the suitable port, and P_m_ is the interference power transmission from the other ports. The insertion loss is given by:

(4) $$Loss_{dB}=-10\log\left(\frac{P_{out}}{P_{in}}\right)\;$$

where P_out_ is the power at the output port, and P_in_ is the power in the input port 1. 

## 3. Results

**Figure 2:**
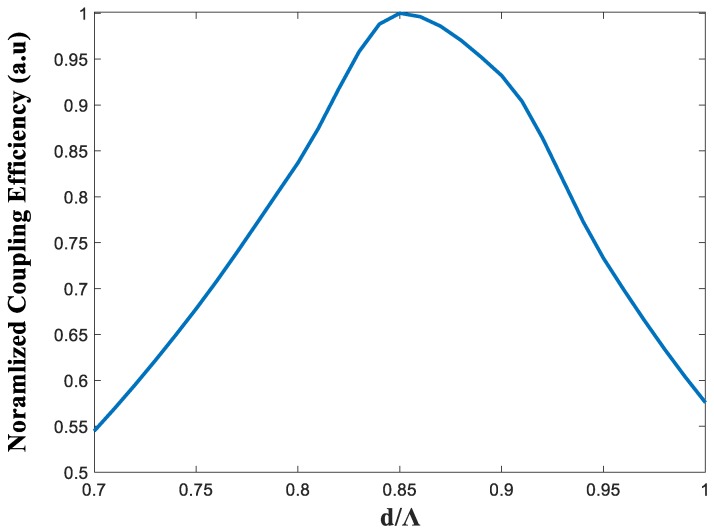
Normalized coupling efficiency as a function of the geometrical parameters of the multicore photonic crystal fiber (PCF) (d/Λ) for the operating wavelengths.

**Figure 3:**
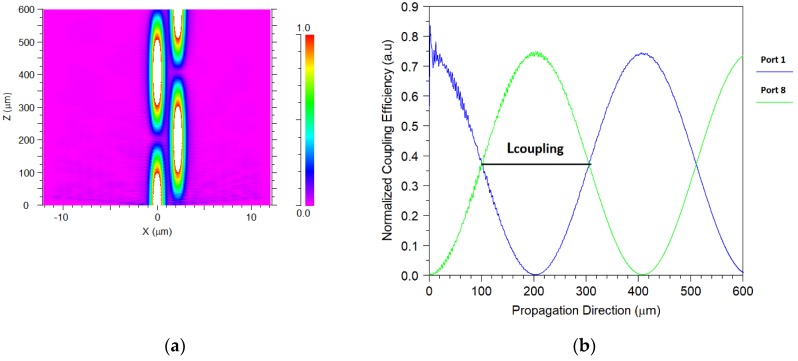
Energy transfer between core 1 and core 8 for the 1565-nm wavelength. (**a**). Intensity profile. (**b**). Normalized coupling efficiency as a function of the propagation direction.

The 1 × 8 PCF wavelength demultiplexer structure was simulated using an RSoft Photonics CAD suite software, which is based on the BPM. Figure 2 shows the optimal geometrical parameters (d/Λ) of the 1 × 8 wavelength demultiplexer multicore PCF structure for the operating wavelengths. It can be noticed from Figure 3 that the optimal value is 0.85 for the operating wavelengths, and the values of d and Λ are 0.97 µm and 1.14 µm.

The coupling length size for each operating wavelength was calculated by simulating the transfer energy between two closer silica cores. Figure 3a,b show the full transfer energy between port 1 and port 8 at the Z-range from 0–600 µm for the 1565–nm operated wavelength. From Figure 3b, it can be noticed that the coupling length value is 203.41 µm. The same simulation was done for each of the operating wavelengths, and the values of the coupling length were found as shown in Table 2. 

By using the results taken from Table 2 and combining them with Equation (2), the locations and the lengths of the silica rods that are suitable for the 1 × 8 wavelength demultiplexer multicore PCF can be found, and their values are: L_main_ = 2.03 cm, L_right,1_ = 0.98 cm, L_left,1_ = 0.96 cm, L_right,2_ = 0.43 cm, L_left,2_ = 0.525 cm, L_right,3_ = 0.485 cm, and L_left,3_ = 0.545 cm. Using these results along with the optimizations over the multicore PCF length, the locations (2.03–2.5cm at the Z-axis in core 1, 3.01–3.445 cm at the Z-axis in core 4, and 3.46–4cm at the Z-axis in core 5) of the three selected switch LiNbO_3_ layers were found. In addition, the eight output port layers were optimized in order to obtain a compact 1 × 8 demultiplexer, and their locations are found as shown in Figure 1a. 

**Figure 4:**
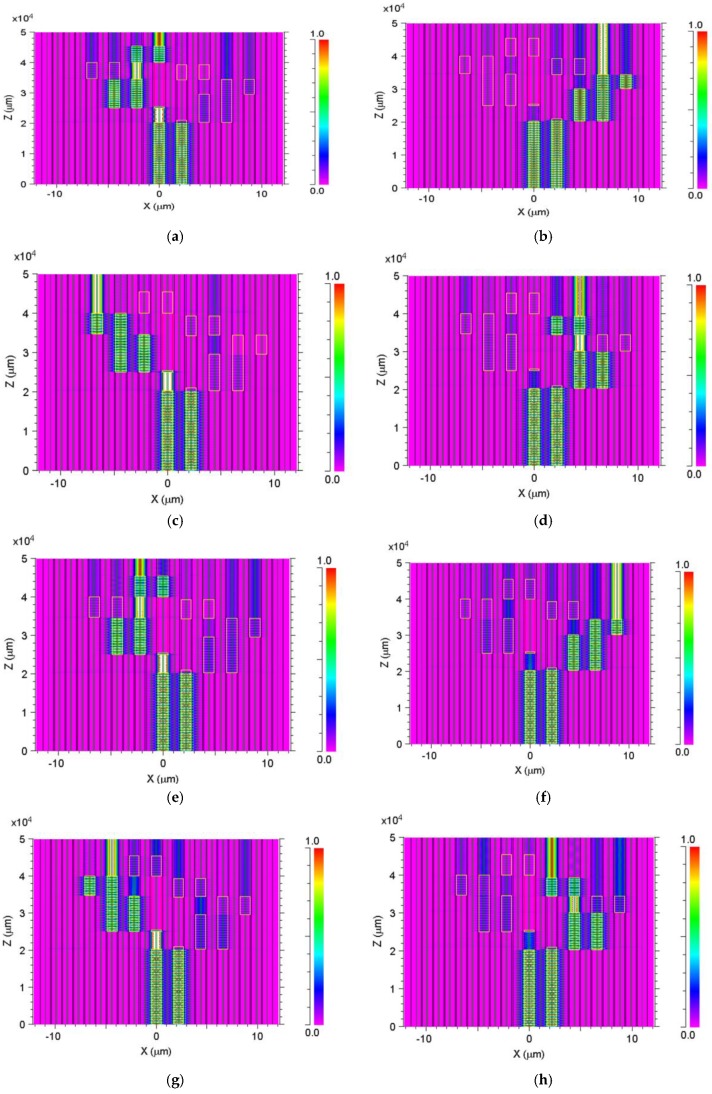
Intensity profile of the 1 × 8 multimode interference (MMI) wavelength demultiplexer: (**a**) λ_1_ = 1530 nm (port 1). (**b**) λ_2_ = 1535 nm (port 2). (**c**) λ_3_ = 1540 nm (port 3). (**d**) λ_4_ = 1545 nm (port 4). (**e**). λ_5_ =1550 nm (port 5). (**f**) λ_6_ = 1555 nm (port 6). (**g**) λ_7_ = 1560 nm (port 7). and (**h**) λ_8_ = 1565 nm (port 8).

Figure 4a shows the light propagation of the 1530-nm wavelength in the PCF structure, and its optical path can be described as follows: z = 0–1.9 cm, light coupled between port 1 and port 8; z = 1.9–2.5 cm, light confined in port 1; z = 2.5–2.6 cm, light coupled from port 1 to port 5; z = 2.6–3.5 cm, light coupled between port 5 and port 7; z = 3.5–4 cm, light confined in port 5; z = 4–4.7 cm, light coupled between port 5 and port 1; and z = 4.7–5 cm, light confined in port 1. Figure 4b shows the light propagation of the 1535-nm wavelength in the PCF structure, and its optical path can be described as follows: z= 0–2cm, light coupled between port 1 and port 8; z = 2–2.15 cm, light coupled from port 8 to port 4; z = 2.15–3 cm, light coupled between port 4 and port 2; z = 3–3.5 cm, light coupled between port 2 and port 6; z = 3.5–5cm, light confined in port 2. Figure 4c shows the light propagation of the 1540-nm wavelength in the PCF structure, and its optical path can be described as follows: z = 0–1.9 cm, light coupled between port 1 and port 8; z = 1.9–2.5 cm, light confined in port 1; z = 2.5–2.6 cm, light coupled from port 1 to port 5; z = 2.6–3.5 cm, light coupled between port 5 and port 7; z = 3.6–4 cm, light coupled between port 3 and port 7; z = 4–5 cm, light confined in port 3. Figure 4d shows the light propagation of the 1545-nm wavelength in the PCF structure, and its optical path can be described as follows: z = 0–2 cm, light coupled between port 1 and port 8; z = 2–2.15 cm, light coupled from port 8 to port 4; z = 2.15–3 cm, light coupled between port 4 and port 2; z = 3–3.5 cm, light confined in port 4; z = 3.5–4 cm, light coupled between port 4 and port 8; and z = 4–5 cm, light confined in port 4. Figure 4e shows the light propagation of the 1550-nm wavelength in the PCF structure, and its optical path can be described as follows: z = 0–1.9 cm, light coupled between port 1 and port 8; z = 1.9–2.5 cm, light confined in port 1; z = 2.5–2.6 cm, light coupled from port 1 to port 5; z = 2.6–3.5 cm, light coupled between port 5 and port 7; z = 3.5–4 cm, light confined in port 5; z = 4–4.7 cm, light coupled between port 5 and port 1; and z = 4.7–5 cm, light confined in port 5. Figure 4f shows the light propagation of the 1555-nm wavelength in the PCF structure, and its optical path can be described as follows: z = 0–2 cm, light coupled between port 1 and port 8; z = 2–2.15 cm, light coupled from port 8 to port 4; z = 2.15–3 cm, light coupled between port 4 and port 2; z = 3–3.5 cm, light coupled between port 2 and port 6; and z = 3.5–5 cm, light confined in port 6. Figure 4g shows the light propagation of the 1560-nm wavelength in the PCF structure, and its optical path can be described as follows: z = 0–1.9cm, light coupled between port 1 and port 8; z = 1.9–2.5 cm, light confined in port 1; z = 2.5–2.6 cm, light coupled from port 1 to port 5; z = 2.6–3.5 cm, light coupled between port 5 and port 7; z = 3.6–4 cm, light coupled between port 3 and port 7; and z = 4–5 cm, light confined in port 7. Figure 4h shows the light propagation of the 1565-nm wavelength in the PCF structure and its optical path can be described as follows: z = 0–2 cm, light coupled between port 1 and port 8; z = 2–2.15 cm, light coupled from port 8 to port 4; z = 2.15–3 cm, light coupled between port 4 and port 2; z = 3–3.5 cm, light confined in port 4; z = 3.5–4 cm, light coupled between port 4 and port 8; and z = 4–5 cm, light confined in port 8.

**Figure 5:**
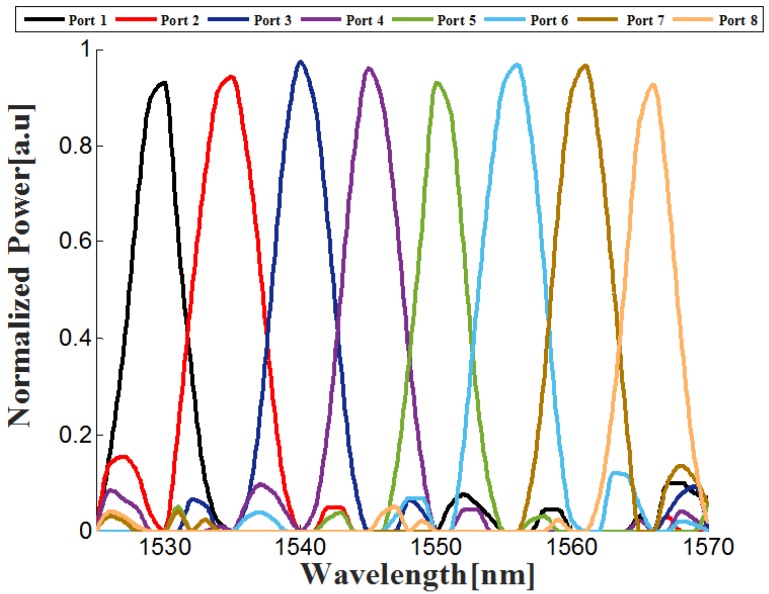
Normalized power as function of the optical signals.

BPM simulations combined with the Matlab script code were performed to determine the 1 × 8 wavelength PCF demultiplexer properties. Figure 5 shows the optical bandwidth transmission results for wavelengths around the C-band range (1530–1565 nm).

From Figure 5, combined with Equations (3) and (4), the values of the cross-talk, insertion losses and full width maximum (FWHM) can be found. Table 3 shows the values of the cross-talk, bandwidth (FWHM), and loss for each port. 

## 4. Conclusions

This study shows how it is possible to implement the classical 1 × 8 demultiplexer that is usually based on seven 1 × 2 demultiplexer units in a cascade tree structure, only with one multicore PCF. In addition, this work shows how to control the light propagation direction inside the multicore PCF using the MTIR and bandage mechanism that enables light to be coupled only in the silica areas, and a strong light confinement in the LiNbO_3_ and Si_3_N_4_ areas. 

By analysis of the numerical results, it is clear that the main benefit is that the multicore PCF was designed without using additional device elements, which are usually required in order for the device to function as a 1 × 8 demultiplexer. This benefit can lead to a new design of a compact DWDM system that can be utilized to obtain better performances. However, integrating this type of demultiplexer to the DWDM system will require modifying the mode field diameter (MFD) coming out from the multicore PCF. This is due to the DWDM system usually using silica fibers that have a large MFD compared to the multicore PCF. This issue can be solved by integrated a tapered fiber that can convert the MFD of the 1 × 8 wavelength demultiplexer to the MFD of the DWDM system. 

The 1 × 8 wavelength demultiplexer multicore PCF can be fabricated using a fiber fabrication facility with hybrid materials. The fabrication process cannot be carried out only by a staking or drawing technique; it also requires a microelectronic technique, such as the lithography technique, that can be used to integrate the LiNbO_3_ and Si_3_N_4_ layers over the multicore PCF length.

To conclude, in this work, we have shown that a 1 × 8 wavelength demultiplexer can be implemented using a multicore PCF structure with integrated LiNbO_3_ and Si_3_N_4_ materials. 

The simulation results showed that the operated wavelengths of 1530 nm, 1535 nm, 1540 nm, 1545 nm, 1550 nm, 1555 nm, 1560 nm, and 1565 mm, with a short spacing of 5 nm and supporting the whole C-band range, can be separated after a propagation length of 5 cm with a bandwidth range of 4.02–4.69 nm.

The proposed device has low cross-talk ((–16.88)–(–15.93) dB) with an insertion loss of 0.18–0.69 dB. 

Thus, this device has the potential to increase the data bitrate in an optical communications system that works on DWDM technology.

This research can be used in future work to design a powerful and new demultiplexer device based on multicore polymer/silica fiber in a very similar way, and with the right materials’ modification.

Although only the 1 × 8 wavelength demultiplexer configuration is considered in this paper, the demultiplexer can also operate as a multiplexer with a reversed direction of the guided light.

## Figures and Tables

**Table 1:** The multicore photonic crystal fiber (PCF) materials’ reflective index values.

λ_m_ (nm)	1530	1535	1540	1545	1550	1555	1560	1565
n_Si_3_N_4__	1.9968	1.9967	1.9966	1.9964	1.9963	1.9961	1.996	1.9959
n_Silica_	1.4443	1.4442	1.4441	1.4441	1.444	1.444	1.4439	1.4438
n_LiNbO_3__	2.1481	2.148	2.1478	2.1477	2.1476	2.1474	2.1473	2.1471

**Table 2:** The coupling length values.

λ_m_ (nm)	1530(λ_1_)	1535(λ_2_)	1540(λ_3_)	1545(λ_4_)	1550(λ_5_)	1555(λ_6_)	1560(λ_7_)	1565(λ_8_)
L_Coupling_(µm)	230.89	225.1	222.83	219.12	214.71	211.11	208.34	203.41

**Table 3:** Values of the cross-talk, full width maximum (FWHM), and losses for the operating wavelengths.

λ_m_ (nm)	1530	1535	1540	1545	1550	1555	1560	1565
Port number	1	2	3	4	5	6	7	8
Cross-talk (dB)	–16.65	–16.73	–16.88	–16.81	–16.6	–16.32	–16.08	–15.93
FWHM (nm)	4.23	4.38	4.67	4.69	4.03	4.1	4.62	4.15
Losses (dB)	0.31	0.26	0.2	0.18	0.55	0.31	0.45	0.69
